# Bioactive Compounds in Rosehip (*Rosa canina*) Powder with Encapsulating Agents

**DOI:** 10.3390/molecules27154737

**Published:** 2022-07-25

**Authors:** Marta Igual, Patricia García-Herrera, Rosa M. Cámara, Javier Martínez-Monzó, Purificación García-Segovia, Montaña Cámara

**Affiliations:** 1 Food Investigation and Innovation Group, Food Technology Department, Universitat Politècnica de València, Camino de Vera s/n, 46022 Valencia, Spain; marigra@upvnet.upv.es (M.I.); xmartine@tal.upv.es (J.M.-M.); 2 Departamento Nutrición y Ciencia de los Alimentos, Facultad de Farmacia, Universidad Complutense de Madrid (UCM), Pza. Ramón y Cajal, s/n, 28040 Madrid, Spain; patrigar@pdi.ucm.es (P.G.-H.); rosacama@ucm.es (R.M.C.)

**Keywords:** rosehip, bioactive compounds, antioxidant capacity, encapsulation

## Abstract

*Rosa canina* pseudo-fruits contain interesting bioactive compounds. This work aims to evaluate the use of different biopolymers as encapsulating agents on the content of organic acids, minerals, fibers, phenols, carotenoids, and the antioxidant activity of the powdered product. Fruits were ground and freeze-dried with or without biopolymers (maltodextrin, resistant maltodextrin, cyclodextrin, and pea protein). Rosehip formulated purees with encapsulating agents are an interesting food ingredient rich in fiber and minerals that could be used in the food industry in order to obtain different functional foods. Results obtained in this study show that all formulated samples are a good source of potassium, calcium, magnesium, and manganese. Both rosehip without biopolymers and rosehip with pea protein formulations are also a good source of Zn. Formulation with pea protein can be claimed as a good source of Fe. All formulations are food ingredients with a very high content of ascorbic acid. Comparing the encapsulating agents, depending on the studied bioactive compound samples behaved differently. In conclusion, it can be indicated that pea protein is recommended as an encapsulating agent since the rosehip with pea protein sample has the highest content of fiber, minerals, organic acids, and carotenoids among the encapsulating agents studied.

## 1. Introduction

**Figure 1:**
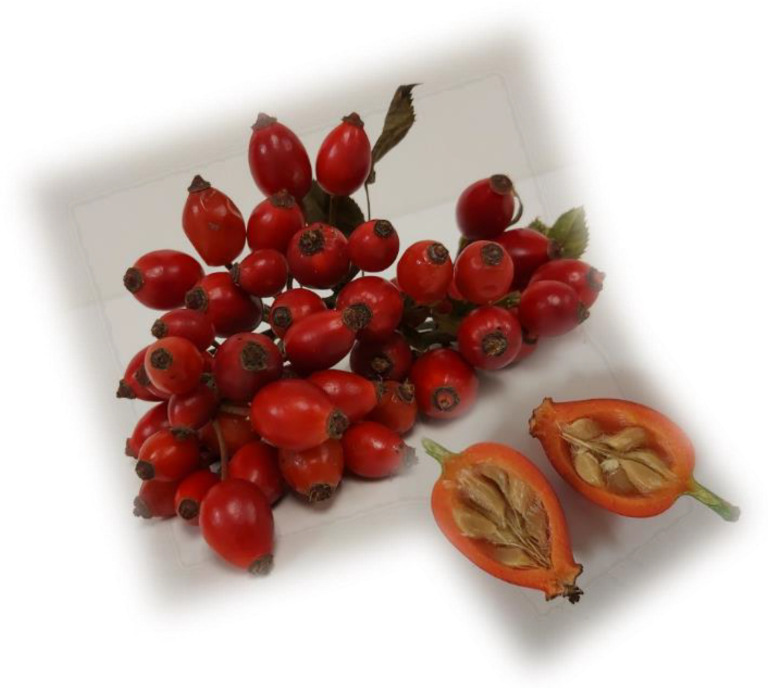
Rosehips of *Rosa canina* and lengthwise section fruit.

Wild rose (*Rosa canina* L.) is a native shrub that belongs to the *Rosaceae* family and is widespread in northern Europe, Asia, the Middle East, and North America. For centuries, the pseudo-fruits of *Rosa canina* (rosehip) have been recognized as valuable food and medicine constituents due to their notable content of pro-health compounds [1]. The beneficial health effects are related to their rich content in flavonoids, carotenoids, fatty acids, and vitamins [2]. However, like other fruits, it is perishable, its production is seasonal, and its consumption is made difficult by the physiology of the pseudo-fruit. The pseudo-fruits, rosehips, are aggregate fruits comprising several achenes enclosed by an enlarged, red, fleshy floral cup (hypanthium) (Figure 1). The medium weight of the fruit is 2.8 to 2.9 g, distributed between the pericarp (65–70%) and the hard and hairy seeds (30–35%) [3,4].

The commercialization of dried fruit-based products can offer solutions to problems related to the short shelf life of fruits and their seasonality and a means of providing microbiologically stable products because of their low water activity. Furthermore, dried fruit both facilitates the shipping operations and makes them more profitable due to its lower volume and weight and easier handling. In the case of rosehips, drying [5] and specifically obtaining powder represents an alternative for its consumption due to the complexity of the use of the fruit, which is limited to infusions or jams. Obtaining a powdered product would encourage its consumption as rehydrated as a juice or infusion, or to be added to desserts, dairy products, salads, ice cream, snacks, among other things, and even for enriching almost any food in bioactive compounds [6,7,8]. 

Freeze-drying is one of the drying methods that provide the highest retention of chemical profile and antioxidant activity in foods, attributed to its less intense heating [9]. In fact, several studies have successfully obtained a vegetable/fruit snack with good physical, chemical, and functional properties by using freeze-drying [10,11,12,13,14]. Moreover, to improve the quality and stability of the freeze-dried fruit, the addition of high molecular weight additives to the product before drying as a carrier and anticaking agents is a widely used alternative to stabilize hygroscopic powders [15,16]. The use of biopolymers improves and maintains the characteristics of powder products but also allows the microencapsulation of bioactive compounds from the matrix. Maltodextrins are usually added during the production of food powders in order to act as encapsulating or wall materials, contributing to keeping the desired functional properties in the finished product [16]. However, there are other encapsulate agents with prebiotic effects as resistant maltodextrins [17] or proteins biopolymers from plants as pea protein [18] as an alternative. In addition, the typical use of cyclodextrins in cosmetics is being extended to food and, therefore, could also be a possibility to be explored [19].

Microencapsulation provides a physical barrier around the microencapsulated compounds, reducing the contact and reactivity of the encapsulated material with the environment [20]. Consequently, microencapsulation has been proved to be an excellent tool for the stabilization of bioactive compounds [21,22] and the inclusion of compounds in food matrices as food ingredients [23]. 

Depending on the final composition, formulated food ingredients rich in bioactive compounds could be of great interest for industry purposes both in functional foods formulation as well as to be used as an ingredient in food supplements [24].

In order to obtain a powdered rosehip product that facilitates its commercialization, this work aims to evaluate the use of different biopolymers as encapsulating agents on the content of organic acids, minerals, fiber, phenols, carotenoids, and the antioxidant activity of the powdered product. 

## 2. Results and Discussion

### 2.1. General Parameters

**Figure 2:**
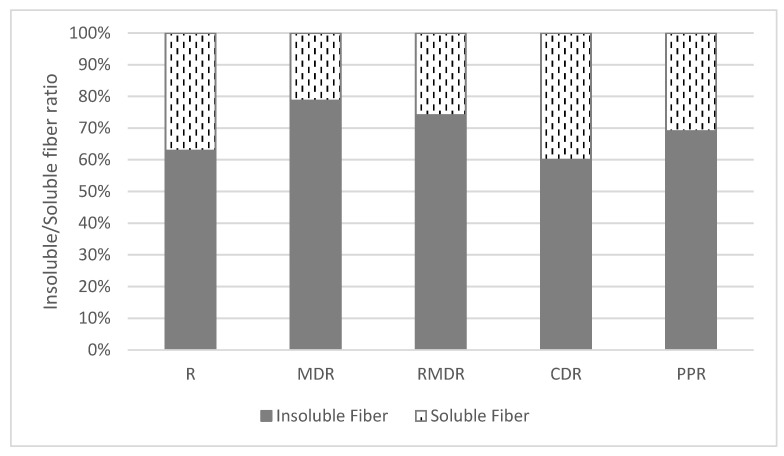
Insoluble/soluble fiber ratio in samples analyzed. R: rosehip; MDR: maltodextrin rosehip; RMDR: resistant maltodextrin rosehip; CDR: cyclodextrin rosehip; PPR: pea protein rosehip.

Table 1 shows the values of Xw, Brix degrees, and pH of the purees obtained according to Figure 2 and as explained in Section 2.2. The water content of the powdered products obtained from each puree is also shown (Table 1). The addition of biopolymers to the rosehip puree reduced significantly (*p* < 0.05) the Xw of the mixtures. Furthermore, the addition of MD, RMD, and PP increased significantly (*p* < 0.05) the Brix degrees of the mixtures and, therefore, the soluble solids, as was observed by other works [17,25]. The pH increased significantly (*p* < 0.05) in the mixtures with CD and PP. The Xw of the powder products obtained showed significant differences (*p* < 0.05). The addition of any biopolymer reduced significantly (*p* < 0.05) the Xw of the powder, but this decrease was more drastic in the samples with PP. Probably PPR presented a greater facility for the water exit from the matrix. Generally, powder samples that come from purees with higher water and/or solutes content presented higher water content after freeze-drying. Water content is related to the drying efficiency, powder flowability, stickiness, and storage stability due to its effect on glass transition and crystallization behavior [26]. In this sense, R would be more susceptible to undesirable physical changes. The use of the biopolymers studied, especially PP, could protect against these changes. This fact was observed by other authors in orange pulp [27]. 

### 2.2. Insoluble, Soluble, and Total Dietary Fiber

Results from insoluble (IF), soluble (SF), and total dietary fiber (TF) are shown in Table 2. Rosehip powdered puree is characterized by its high IF content, accounting for more than 60% of the TF (Figure 2). Formulation of rosehip with starch derivates results in a decrease in TF as well in IF and SF fractions. Even with lower content, CDR formulation keeps the same ratio IF:SF as rosehip R. 

It is important to highlight the significant decrease in SF on MDR and RMDR samples in which the IF represents almost 80% of total fiber. In the case of the rosehip puree formulated with pea protein, the PPR sample, fiber content, and profile are more similar to the original R puree. That means that the most significant changes, a reduction in fiber content, occur in starch derivates formulated samples.

Fiber consumption in Western societies is insufficient, and its deficiency is directly linked to certain diseases. The required daily fiber intake can be obtained from foods such as fruits and vegetables, whole grains, legumes, nuts, and others, or by eating foods enriched with fiber as a functional ingredient [28]. Results from our study show that all the formulated samples analyzed can be considered valuable ingredients with to be used in the food industry for food products fiber enrichment as all formulations can be classified as “high fiber content” according to Regulation 1169/2011 and Regulation 1924/2006 [29,30].

### 2.3. Mineral Content

Respecting the mineral content, potassium is the main microelement, and manganese is the main microelement found in all formulated samples, as expected for vegetable samples (Table 3). It is important to note that Na was not quantified in any of the analyzed samples (Na limit of detection (LOD) = 0.394 ppm and quantification (LOQ) = 1.314 ppm). The rosehip sample (R) showed higher macro and microelements content than other rosehip formulations with starch derivates as encapsulating agents. Iron content in R is lower (0.24 mg/100 g) than the content reported by other authors [31,32,33], who found 5.69, 1.22, and 2.34 mg/100 g, respectively. Manganese values for the rosehip sample are, according to other authors [32,34] although higher than values (1.46–3.20 mg/100 g, respectively) reported by [33,35]. Within the encapsulating agents studied, the PPR sample presented a higher potassium and magnesium content than the rest of the formulations due to its plant origin and pea minerals contribution. Potassium content in all samples is higher than expected, as values reported by [34,35], who studied the potassium content in rose canine, were in the range of 914–944 mg/100 g. Looking for the relations between minerals, the calcium/magnesium ratio was favorable to calcium in all samples studied, being the lower calcium content for PPR. Ratio Fe/Cu always is higher than 1 in all samples. Further, all samples presented a ratio of Mn/Zn favorable for Mn as it would be expected for wild fruits [36].

According to labeling regulation 1924/2006, it can be said that a food is a “source of [mineral]” or “high content of [mineral]” when it covers 15% and 30%, respectively, of the nutrient reference values. All formulated samples could be marketed as a good source of potassium, calcium, magnesium, and manganese. Both R and PPR formulations are also good sources of Zn, and PPR is the only one that can be claimed as a good source of Fe (6.05 mg/100 g). 

### 2.4. Bioactive Compounds and Antioxidant Capacity

Results from bioactive compounds analyzed in formulated samples, such as organic acids, total polyphenols, and carotenoids, are shown in Table 4 and Table 5.

**Figure 3:**
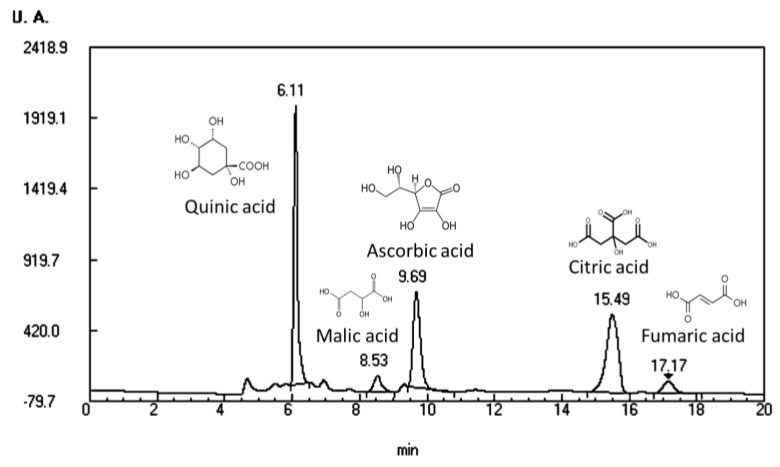
–HPLC-UV organic acid profile of rosehip sample.

Regarding the organic acids, Figure 3 and Table 4 show the organic acids profile and content on analyzed samples. As expected, the rosehip R sample is the one with a higher organic acid content, being citric acid the predominant. This disagrees with other authors who found malic or ascorbic acid as the majority organic acid for rosehip [37,38], but it agrees with [39], who reported malic and citric acid as the main organic acids in fruits of rosehip fruits. Nevertheless, the ascorbic acid content in our samples is inside the range (0.2–0.85 g/100 g) reported by [40].

Rosehip fruit is characterized by its high ascorbic acid content. In our study, the ascorbic acid decrease could be due to the obtaining process of the powder by the high temperatures or light exposition. With the independence of that, all formulated samples analyzed can be considered as very valuable food ingredients and claimed as “high ascorbic acid content”, according to Regulation 1169/2011; thus, these ingredients could be considered as and be of great interest for the food industry. As expected, PPR organic acid profile is different from starch derivate formulations due to the pea protein contribution to the organic acid content of the final formulation. 

Table 5 shows the total content of carotenoids and phenols mean values and standard deviations in brackets, as well as the antioxidant capacity of the powdered products. It can be observed that the bioactive compounds content studied or antioxidant capacity is higher in R compared to the rest samples. This fact is due to all R content being rosehips and providing these bioactive compounds and the rest of the samples presenting 48% of biopolymers in their composition. 

Depending on the studied bioactive compound, samples behaved differently. In the case of carotenoids, the use of PP allowed obtaining a rosehip powder product with a higher content of them; however, the content of phenolic compounds is higher in the rosehip sample encapsulated with MD. This behavior probably will be related to the affinity of encapsulation agents with the bioactive compounds. Maltodextrins are usually employed to encapsulate hydrosoluble compounds such as ascorbic acid, phenolic compounds, or fruit juices, and proteins are used to encapsulate liposoluble substances such as lycopene or polyunsaturated fatty acids [41]. In the case of CDR, the TP value was significantly (*p* < 0.05) the lowest, mainly due to cyclodextrin conformation. They are shaped like a conical toroid with the secondary OH groups (corresponding to carbons 2 and 3 of glucose) on the widest face, the primary OH groups (corresponding to carbon 6) on the opposite face, and the Hs oriented inward of the cavity. Therefore, they have a hydrophobic cavity and a hydrophilic outer part. This fact makes them capable of forming inclusion complexes with essentially apolar molecules and of a suitable size for the host–guest interaction [19]. In this way, the cyclodextrin would be easier to encapsulate and protect TC than TP. As can be seen in Table 5, the TC values for CDR are the next highest, after PPR, in the samples containing biopolymers. AC values showed the same trend as TP values; in addition, the Pearson coefficient between AC-TP was 0.9973 (*p* < 0.05); therefore, they are highly correlated, and TP presents an important role in the AC of studied products.

**Figure 4:**
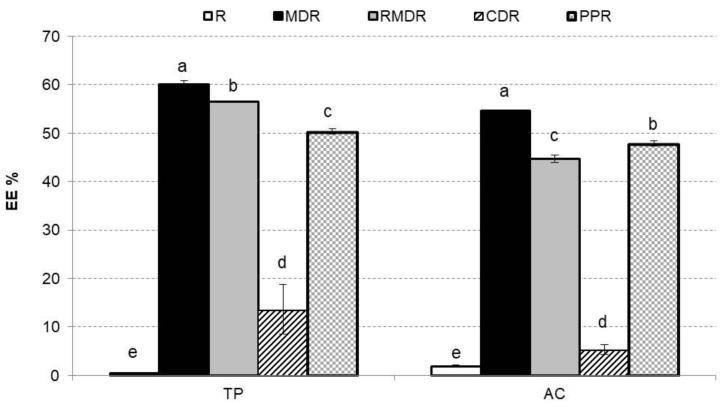
Mean values and standard deviation of encapsulation efficiencies percentage in rosehip freeze-dried formulated samples for total phenols and antioxidant capacity. Letters indicate homogeneous groups established by the ANOVA (*p* < 0.05) for total phenols and antioxidant capacity. R: rosehip; MDR: maltodextrin rosehip; RMDR: resistant maltodextrin rosehip; CDR: cyclodextrin rosehip; PPR: pea protein rosehip.

**Figure 5:**
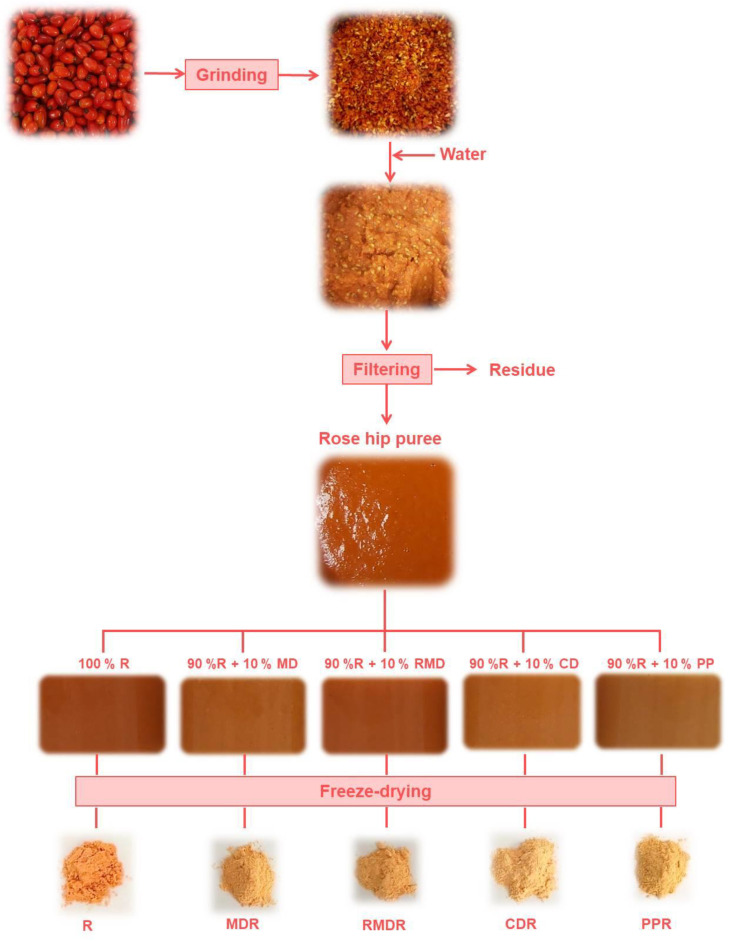
Scheme of rosehip powders obtaining.

Figure 4 shows TP and AC EE % in rosehip freeze-dried samples. EE refers to the potential of the wall material to encapsulate or hold the core material inside the microcapsule [42]. EE is also related to the shelf life of the phenolic compounds content and AC in the powder. In the first place, the practically null EE% values of R (TP and AC) stand out since it does not contain biopolymers in its composition, and there is no encapsulation. Low EE% CDR values can also be observed, reaching only 14% for TP and 5% for AC. As indicated above, the conformation of the cyclodextrin generates a cavity in which to stay the molecules to be encapsulated, but these molecules must be hydrophobic to be linked and carry out the host–guest interaction [19]. Figure 5 evaluates the EE% for TP that are hydrophilic compounds. MDR, RMDR, and PPR presented EE for TP from 50% to 60% and for AC from 45% to 55%, values similar to those found in references for other vegetable products [18,43]. The sample with the highest EE % values was MDR. 

## 3. Materials and Methods

### 3.1. Raw Materials

Rosehip (*R. canina*) fruits were manually harvested in Aldehuela (Teruel, Spain) in October 2020. Maltodextrin (GLUCIDEX^®^ 12) (MD), pea protein powder (Nutralys^®^ S85F) (PP), and beta cyclodextrin (KLEPTOSE^®^) (CD) were supplied by Roquette S.L. (Valencia, Spain). Resistant maltodextrin (Fibersol-2^®^) (RMD) was purchased from ADM/Matsutani, LLC (Decatur, IL, USA).

### 3.2. Sample Preparation 

Figure 5 shows a scheme of powder processing. Rosehips (1000 g) were washed with distilled water and homogenized within a Thermomix (TM 21, Vorwerk, Valencia, Spain) for 1 min at 5200 rpm. Then, distilled water (1000 g) was added and newly re-homogenized for 5 min at 5200 rpm. The mixture was filtered using a sieve (light of mesh diameter 1 mm, Cisa 029077, 1 series). Four different formulations were prepared by adding 10 g of MD, RMD, CD, or PP to 90 g of the filtered mixture. Moreover, a control sample (R) without biopolymers was prepared. Five purees were as follows: R (100% rosehip), MDR (10% maltodextrin: 90% rosehip), RMDR (10% resistant maltodextrin: 90% rosehip), CDR (10% cyclodextrin: 90% rosehip), and PPR (10% pea protein: 90% rosehip). Then, the control rosehip and formulated purees were freeze-dried. A puree layer (0.5 cm thickness) was placed in a standardized aluminum plate (15 cm diameter and 5 cm height). Consecutively, samples were stored at −45 °C (Vertical Freezer, CVF450/45, Ing. Climas, Barcelona, Spain) for 24 h before being dried in a Lioalfa-6 Lyophyliser (Telstar, Spain) at 2600 Pa and −56.6 °C for 48 h. The freeze-dried samples were ground in a grinder (Minimoka, Taurus, Lleida, Spain) to obtain a free-flowing powder. 

### 3.3. Analytical Determinations

#### 3.3.1. Water Content, Degree Brix, and pH

Water content (Xw, grams water per 100 g of product), degree Brix (grams soluble solids per 100 g liquid phase), and pH were determined for the control and formulated purees. Water content was also determined in powder samples. The Xw was determined by drying the sample to a constant weight at 70 °C in a vacuum oven [44]. Degree Brix was measured in previously homogenized samples with a refractometer at 20 °C (Abbemat 200, Anton Paar, Austria). pH determination of purees was made using a Basic 20 pH meter (Crison, Spain). All determinations were performed in triplicate.

#### 3.3.2. Fiber

Total, soluble, and insoluble fiber were determined by AOAC 991.43 enzymatic gravimetric method [45]. The first samples were under enzymatic digestion with α-amylase, protease, and amyloglucosidase (Sigma-Aldrich, St. Louis, MO, USA) in order to eliminate the protein and starch present in the samples. To obtain the insoluble fiber, we proceeded to the filtrate of the liquid obtained on crucibles with a Gooch Pyrex filter plate, and later, they were dried in an oven at 100 °C and then weighed. The reserved liquid of the insoluble fiber was stored in a 500 mL flask with the addition of 400 mL of 96% *v*/*v* ethanol and precipitated from one day to the next. Next, it was filtered in new Gooch Pyrex crucibles with the same insoluble fiber conditions. In both cases, the content of protein and ash was determined, and the content corresponding to insoluble and soluble fiber was calculated, respectively.

#### 3.3.3. Ash Content and Mineral Composition

Method 930.05 of AOAC for ash determination was used [45]. Incineration was performed in an oven (Muffle MR 170, W.C. Heraeus Hanau, Hanau, Germany) for 24 h at 550 C, and ashes were gravimetrically quantified. The residue was extracted with HCl (50% *v*/*v*) and HNO_3_ (50% *v*/*v*) to measure Fe, Cu, Mn, and Zn and was directly quantified. To avoid interferences between different elements, a dilution with 1.16 La_2_O_3_/100 HCl (resulting LaCl_2_) was performed to analyze Ca and Mg and with CsCl (0.2 g/100 g solution) to analyze Na and K. All measurements were performed in atomic absorption spectroscopy (AAS) in Analyst 200 Perkin Elmer equipment (Perkin Elmer, Waltham, MA, USA), comparing absorbance responses with analytical standard solutions for AAS.

#### 3.3.4. Organic Acids

Organic acids were determined based on protocols described by Sánchez-Mata et al. [46]. Extraction was performed with 0.5 g of sample in 25 mL of 3% m-phosphoric acid and analyzed using an HPLC-UV methodology. The HPLC equipment used was a liquid chromatograph (Micron Analítica, Madrid, Spain) equipped with a Sphereclone ODS (2) 250 * 4.60 mm, 5 µm Phenomenex column, isocratic pump (model PU-II), an AS-1555 automatic injector (Jasco, Tokyo, Japan), and a UV-visible detector (Thermo Separation Spectra Series UV100, Waltham, MO, USA), 215 nm for organic acids. The mobile phase was 1.8 mM H_2_SO_4_ (pH = 2.6), with a flow rate of 0.4 mL/min for organic acids, and injection volume was 100 µL for samples and serial volumes for the standard curve (20, 30, 40, 50, 60, 70, 80, 90, and 100 µL). The compounds were identified by chromatographic comparisons with authentic standards (quinic (0.152 mg/mL), ascorbic (0.155 mg/mL), malic (0.403 mg/mL), fumaric (0.254 mg/mL) and citric acids (0.307 mg/mL), all from Sigma, St. Louis, MO, USA), using linear calibration curves of all compounds for quantification purposes. All data were analyzed using Biocrom 2000 3.0 software (Biocrom, Madrid, Spain).

#### 3.3.5. Total Phenols (TP)

Determining TP was based on the Folin–Ciocalteu method. Briefly, 1 g of sample was mixed with 5 mL methanol, 0.5 mL HCl 5N, NaF 2 mM and centrifugated at 12,857× *g*, 4 °C, 10 min using an Eppendorf centrifuge (Eppendorf, Hamburg, Germany). From the supernatant, 250 µL were mixed in a 25 mL volumetric flask with 1.25 mL Folin–Ciocalteu reagent and stored in a darker place for 8 min. Afterward, 3.75 mL Na_2_CO_3_ with a concentration of 7.5% was added and further stored for 120 min [47]. Absorbance was measured at 765 mm in a UV-3100PC spectrophotometer (VWR, Leuven, Belgium). The total phenolic content was expressed as mg of gallic acid (Sigma-Aldrich, Steinheim, Germany) equivalents (GAE) per 100 g.

#### 3.3.6. Total Carotenoids (TC)

The TC in the samples (1 g) was extracted with a solvent hexane/acetone/ethanol mixture (50:25:25, *v*/*v*/*v*) for 30 min following the Olives et al. [48] method in triplicate. The absorbance of the hexane layer of the sample extracts was measured at 446 nm in a UV-visible spectrophotometer (Thermo Electron Corporation). The TC content was expressed as mg of β-carotene (Fluka-Biochemika, Buchs, Switzerland) per 100 g.

#### 3.3.7. Antioxidant Capacity (AC)

AC was assessed using the free radical scavenging activity of the samples evaluated with the stable radical 2,2-diphenyl-1-picryl-hydrazyl-hydrate (DPPH) following Igual et al. [14] methodology in triplicate. Samples were mixed with methanol. The homogenate was centrifuged (12,857× *g*, 10 min, 4 °C) to obtain the supernatant. A total of 0.1 mL of supernatant was added to 3.9 mL of DPPH (0.030 g/L, Sigma-Aldrich, Steinheim, Germany) in methanol. A UV-visible spectrophotometer (Thermo Electron Corporation) was used at the absorbance at 515 nm. The results were expressed as milligram Trolox equivalents (TE) per 100 g.

#### 3.3.8. Encapsulation Efficiencies (EE) 

To evaluate the EE, analyzed total phenols or antioxidant capacity in each case (TP or AC) content, represented as TB, and surface analyzed bioactive compounds (SB) content of the samples were determined after freeze-drying [18]. For TB determination, samples were treated according to TP or AC. For SB determination, samples were not ground to destroy microcapsules. Only samples were extracted with the solvents in a vortex for 30 s and filtered through a 0.45 µm-size filter following the procedure of [42]. The % EE was calculated using the following Equation (1), where EE refers to encapsulation efficiencies, TB refers to total phenols or antioxidant capacity content, and SB to surface analyzed bioactive compounds.

(1) $$\%\;\mathrm{EE}=\frac{\left(\mathrm{TB}-\mathrm{SB}\right)}{\mathrm{TB}}\times 100$$

### 3.4. Statistical Analysis

Analysis of variance (ANOVA) was applied with a confidence level of 95% (*p* < 0.05) to evaluate the differences among samples. Statgraphics Centurion XVII Software, version 17.2.04 (Statgraphics Technologies, Inc., The Plains, VA, USA) was used. The method used to discriminate between means is Fisher’s least significant difference procedure.

## 4. Conclusions

Rosehip formulated purees with encapsulating agents are an interesting food ingredient rich in fiber and minerals that could be used in the food industry in order to obtain different functional foods. All formulated samples could be marketed as good sources of potassium, calcium, magnesium, and manganese. Both R and PPR formulations are also good sources of Zn, and PPR is the only one that can be claimed as a good source of Fe. In addition, all formulations can be considered food ingredients with a very high content of ascorbic acid. Comparing the encapsulating agents, depending on the studied bioactive compound samples behaved differently. In the case of carotenoids, the use of PP allowed obtaining a rosehip powder product with a higher content of them; however, the content of phenolic compounds is higher in the rosehip sample encapsulated with MD. In general, we can conclude that pea protein is recommended as an encapsulating agent since the PPR formulations have the highest content of bioactive compounds: fiber, minerals, organic acids, and carotenoids, among the encapsulating agents studied. 

## Figures and Tables

**Table 1:** Mean values (and standard deviations) of water content in g water/100 g (Xw puree), Brix degree and pH of puree, and water content in g water/100 g (Xw powder) of formulated rose hip purees and powders.

	Puree Samples			Freeze-Dried Samples
Sample	Xw Puree	Brix Degrees	pH	Xw Powder
R	88.00 (0.06) ^a^	11.87 (0.20) ^c^	3.76 (0.02) ^c^	2.48 (0.01) ^a^
MDR	80.08 (0.02) ^c^	21.27 (0.22) ^a^	3.78 (0.02) ^c^	2.16 (0.08) ^b^
RMDR	79.73 (0.03) ^d^	21.17 (0.21) ^a^	3.76 (0.02) ^c^	2.15 (0.04) ^b^
CDR	80.34 (0.12) ^b^	12.00 (0.20) ^c^	3.84 (0.02) ^b^	1.80 (0.07) ^c^
PPR	80.38 (0.26) ^b^	16.10 (0.20) ^b^	4.57 (0.02) ^a^	0.86 (0.03) ^d^

The same letter in superscript within column indicates homogeneous groups established by ANOVA (*p* < 0.05). R: rosehip; MDR: maltodextrin rosehip; RMDR: resistant maltodextrin rosehip; CDR: cyclodextrin rosehip; PPR: pea protein rosehip.

**Table 2:** Mean values (and standard deviations) of insoluble, soluble, and total fiber content of rosehip formulated samples (g/100 g).

Sample	Insoluble Fiber	Soluble Fiber	Total Fiber
R	17.12 (0.26) ^d^	10.11 (0.00) ^e^	27.13 (0.09) ^d^
MDR	12.61 (0.53) ^c^	3.42 (0.34) ^b^	16.03 (0.55) ^b^
RMDR	8.03 (0.65) ^a^	2.81 (0.06) ^a^	10.35 (0.75) ^a^
CDR	10.02 (0.46) ^b^	6.68 (0.19) ^c^	16.70 (0.57) ^b^
PPR	17.86 (0.66) ^d^	8.01 (0.06) ^d^	25.87 (0.66) ^c^

The same letter in superscript within column indicates homogeneous groups established by ANOVA (*p* < 0.05). R: rosehip; MDR: maltodextrin rosehip; RMDR: resistant maltodextrin rosehip; CDR: cyclodextrin rosehip; PPR: pea protein rosehip.

**Table 3:** Mean values (and standard deviations) of main macro and microelements (mg/100 g) in formulated rosehip samples.

**Macroelements**				
**Sample**	**Na**	**K**	**Ca**	**Mg**
R	nd	1733.5 (19.1) ^d^	534.5 (15.8) ^b^	257.0 (3.2) ^c^
MDR	nd	1167.8 (8.0) ^a^	331.0 (10.1) ^a^	184.7 (1.6) ^a^
RMDR	nd	1307.7 (72.3) ^bc^	329.2 (2.8) ^a^	183.5 (2.0) ^a^
CDR	nd	1241.9 (69.8) ^ab^	317.2 (8.8) ^a^	187.9 (2.9) ^a^
PPR	nd	1356.8 (11.2) ^c^	316.7 (1.7) ^a^	217.2 (6.1) ^b^
**Microelements**				
**Sample**	**Mn**	**Cu**	**Fe**	**Zn**
R	7.39 (0.36) ^c^	0.74 (0.00) ^b^	0.24 (0.02) ^a^	2.16 (0.12) ^c^
MDR	4.37 (0.64) ^a^	0.59 (0.07) ^ab^	1.78 (0.27) ^c^	0.54 (0.03) ^a^
RMDR	5.33 (0.16) ^b^	0.39 (0.02) ^a^	1.02 (0.03) ^b^	1.43 (0.09) ^b^
CDR	4.96 (0.07) ^ab^	0.51 (0.01) ^ab^	0.62 (0.02) ^ab^	1.23 (0.14) ^b^
PPR	5.04 (0.42) ^ab^	1.52 (0.17) ^c^	6.05 (0.43) ^d^	3.68 (0.26) ^d^

The same letter in superscript within column indicates homogeneous groups established by ANOVA (*p* < 0.05). R: rosehip; MDR: maltodextrin rosehip; RMDR: resistant maltodextrin rosehip; CDR: cyclodextrin rosehip; PPR: pea protein rosehip.

**Table 4:** Mean values (and standard deviations) of main organic acids (g/100 g).

Sample	Quinic	Malic	Ascorbic	Citric	Fumaric
R	0.68 (0.01) ^d^	1.55 (0.07) ^d^	0.44 (0.02) ^d^	6.05 (0.24) ^c^	0.175 (0.001) ^b^
MDR	0.50 (0.00) ^b^	1.07 (0.01) ^ab^	0.25 (0.02) ^a^	4.08 (0.06) ^ab^	0.171 (0.008) ^ab^
RMDR	0.49 (0.01) ^b^	1.10 (0.04) ^b^	0.37 (0.02) ^c^	4.08 (0.13) ^ab^	0.172 (0.003) ^ab^
CDR	0.45 (0.04) ^a^	0.98 (0.07) ^a^	0.37 (0.02) ^c^	3.74 (0.41) ^a^	0.166 (0.003) ^ab^
PPR	0.58 (0.02) ^c^	1.26 (0.05) ^c^	0.31 (0.03) ^b^	4.24 (0.08) ^b^	0.165 (0.003) ^a^

The same letter in the superscript within column indicates homogeneous groups established by ANOVA (*p* < 0.05). R: rosehip; MDR: maltodextrin rosehip; RMDR: resistant maltodextrin rosehip; CDR: cyclodextrin rosehip; PPR: pea protein rosehip.

**Table 5:** Mean values (and standard deviations) of total carotenoids (TC), total phenols (TP) content, and antioxidant capacity (AC).

Sample	TC (mg_β__-carotene_/100 g)	TP (mg_GAE_/100 g)	AC (mg_TE_/100 g)
R	74.4 (0.2) ^a^	2482 (8) ^a^	1793 (9) ^a^
MDR	24.43 (0.03) ^d^	1275 (4) ^b^	955 (7) ^b^
RMDR	24.32 (0.12) ^d^	1220 (4) ^c^	928 (4) ^c^
CDR	28.4 (0.2) ^c^	628 (8) ^e^	607 (13) ^e^
PPR	45.9 (0.2) ^b^	799 (4) ^d^	712 (10) ^d^

The same letter in superscript within column indicates homogeneous groups established by ANOVA (*p* < 0.05). R: rosehip; MDR: maltodextrin rosehip; RMDR: resistant maltodextrin rosehip; CDR: cyclodextrin rosehip; PPR: pea protein rosehip.

## Data Availability

Not applicable.

## Sample Availability

Samples of the compounds are not available from the authors.

## Abbreviations

R	Rose hips
MD	Maltodextin
RMD	Resistant maltodextin
CD	Ciclodextrin
PP	Pea protein powder
MDR	90% MD + 10% R
RMDR	90% RMP + 10% R
CDR	90% CD + 10% R
PPR	90% PP + 10% R
TP	Total phenols
GAE	Gallic acid equivalent
TC	Total carotenoids
AC	Antioxidant capacity
DPPH	2,2-diphenyl-1-picryl-hydrazyl-hydrate
TE	Trolox equivalent
EE	Encapsulation efficiencies
TB	Total phenols or Antioxidant Capacity content
SB	Surface analyzed bioactive compounds
